# An Unexpected Dupilumab Reaction

**DOI:** 10.7759/cureus.100629

**Published:** 2026-01-02

**Authors:** Gabriela E Beraja, Connor J Stonesifer, Danyelle M Dawes, Jonette E Keri

**Affiliations:** 1 Dr. Phillip Frost Department of Dermatology and Cutaneous Surgery, University of Miami Miller School of Medicine, Miami, USA; 2 Dermatology, Bruce W. Carter Department of Veterans Affairs Medical Center, Miami, USA

**Keywords:** biologic therapy, crusted scabies, dupilumab, ivermectin, th2 cytokines

## Abstract

Crusted scabies (CS), a rare and highly contagious variant of scabies, is characterized by the uncontrolled proliferation of *Sarcoptes scabiei* and can involve thousands of mites per patient. It often mimics hyperkeratotic disorders, complicating its diagnosis, and extended or repeat courses of treatment are often needed for severe cases. Previous reports have described cases of CS occurring after biologic therapy, with some resolving rapidly following ivermectin treatment. In contrast, our case report includes a patient with CS on dupilumab who required a seven-day extended regimen for complete resolution, which has not been previously reported. Dupilumab’s suppression of Th2 responses may impair the immune system's ability to control scabies mites, allowing unchecked proliferation. This case report contributes to the literature by providing new insights and highlights the potential for persistent scabietic infection in patients receiving dupilumab, underscoring the need for prolonged anti-scabietic treatment in such cases. Further research is needed to explore the immune mechanisms behind the development of CS in patients on immunosuppressive therapies.

## Introduction

Crusted scabies (CS), previously known as Norwegian scabies or scabies crustose, is a highly contagious rare variant of ordinary scabies (OS) characterized by the uncontrolled proliferation of the ectoparasitic mite called *Sarcoptes scabiei* var. *hominis* [1,2]. This occurs when impaired cell-mediated immunity permits unchecked mite replication, further amplified by minimal pruritus and severe hyperkeratosis that allow mites to accumulate in massive numbers [2]. First described by Danielssen and Boeck in 1848 in a Norwegian patient with Hansen’s disease, CS differs from OS in that it can involve thousands to millions of mites per patient [3]. It is characterized by erythematous, hyperkeratotic, and fissured plaques with yellow-to-brown crust. It can occasionally mimic hyperkeratotic disorders such as psoriasis [4], seborrheic dermatitis, Darier’s disease, and palmoplantar keratoderma [5].

Scabies lacks a standardized diagnostic method, but widespread itching, lesions in typical areas, and a household member with similar symptoms strongly suggest the condition [6]. To effectively treat a CS infestation, a combination approach is required. This includes applying either a fully-body application of 5% topical permethrin cream for seven days and then twice a week until clear or 25% topical benzyl benzoate. Additionally, oral ivermectin should be taken at a dose of 200 ug/kg body weight on days 1, 2, 8, 9, and 15, totaling five doses. For severe cases, a seven-day regimen may be necessary, with additional treatment on days 22 and 29 [7,8]. Oral ivermectin is not Food and Drug Administration (FDA)-approved for scabies, but it is included in the World Health Organization’s essential medicines list, recommended in Centers for Disease Control and Prevention (CDC) guidelines, and in the European Academy of Dermatology and Venereology 2017 guidelines [9,10]. Multiple case reports support its efficacy [7,11,12].

A clinical grading scale assists in managing CS, with higher scores indicating a greater disease burden that necessitates more frequent ivermectin doses [13]. Using this scale, Hasan et al. confirmed that patients with Grade 3 severity treated with a median of seven doses of oral ivermectin had a higher proportion of negative skin scraping results (86%) compared to those with Grade 1 (60%) and Grade 2 (72%) severity who received three-day and five-day regimens, respectively. This emphasizes the need for a prolonged and aggressive treatment approach for effective mite eradication [14].

CS typically arises in the setting of severe immune deficiency either from disease or immunosuppressive therapy and may also occur in patients with reduced sensation, limited mobility, or genetic susceptibility [10]. A systematic review of 683 case reports of CS from January 1998 to July 2023 found that 21.4% of cases were associated with immunosuppressive risk factors (e.g., 10.2% association with HIV/AIDS and 8.5% with human T-lymphotropic virus (HTLV)-1) and 10.5% were overtly immunocompetent with no known risk factors. Notably, 2% were related to targeted immunotherapy, such as biologics [15].

Because CS can present insidiously with minimal pruritus and can progress to severe bacterial superinfections, the condition poses a particular danger in immunosuppressed patients [2]. A recent systematic review identified 10 cases of CS following biologic therapy, including two patients treated with dupilumab; one for atopic dermatitis, who received a five-day regimen of ivermectin, and another for ulcerative colitis, who was treated with a two-day regimen [16].

Here, we present an additional case of CS in a patient on dupilumab that required a seven-day extended regimen for complete resolution, which has not been previously reported.

## Case presentation

**Figure 1:**
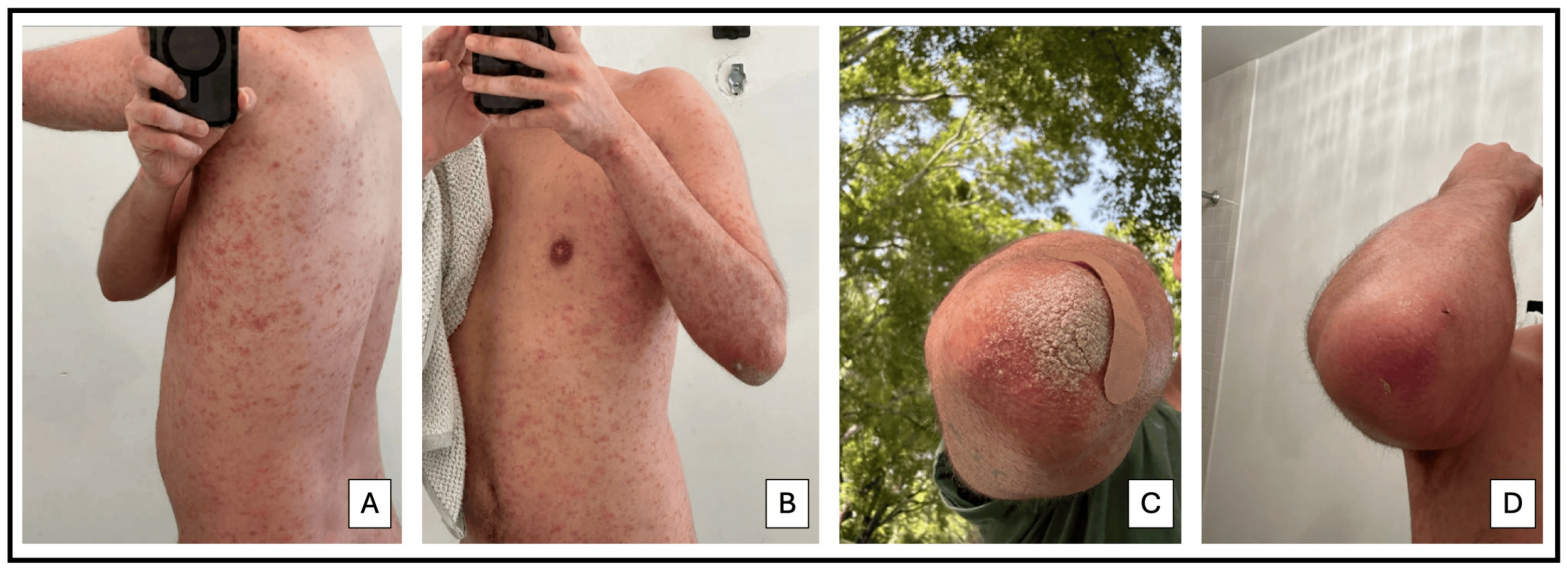
Clinical Progression of Skin Findings A: Multiple red-orange papules and plaques with overlying scale on the upper extremities and trunk. B: Numerous red-orange, scaly papules and plaques distributed across the upper limbs and trunk. C: Well-circumscribed yellow-orange, hyperkeratotic plaque on the right elbow on an erythematous and macerated base. D: Resolution following treatment.

**Figure 2:**
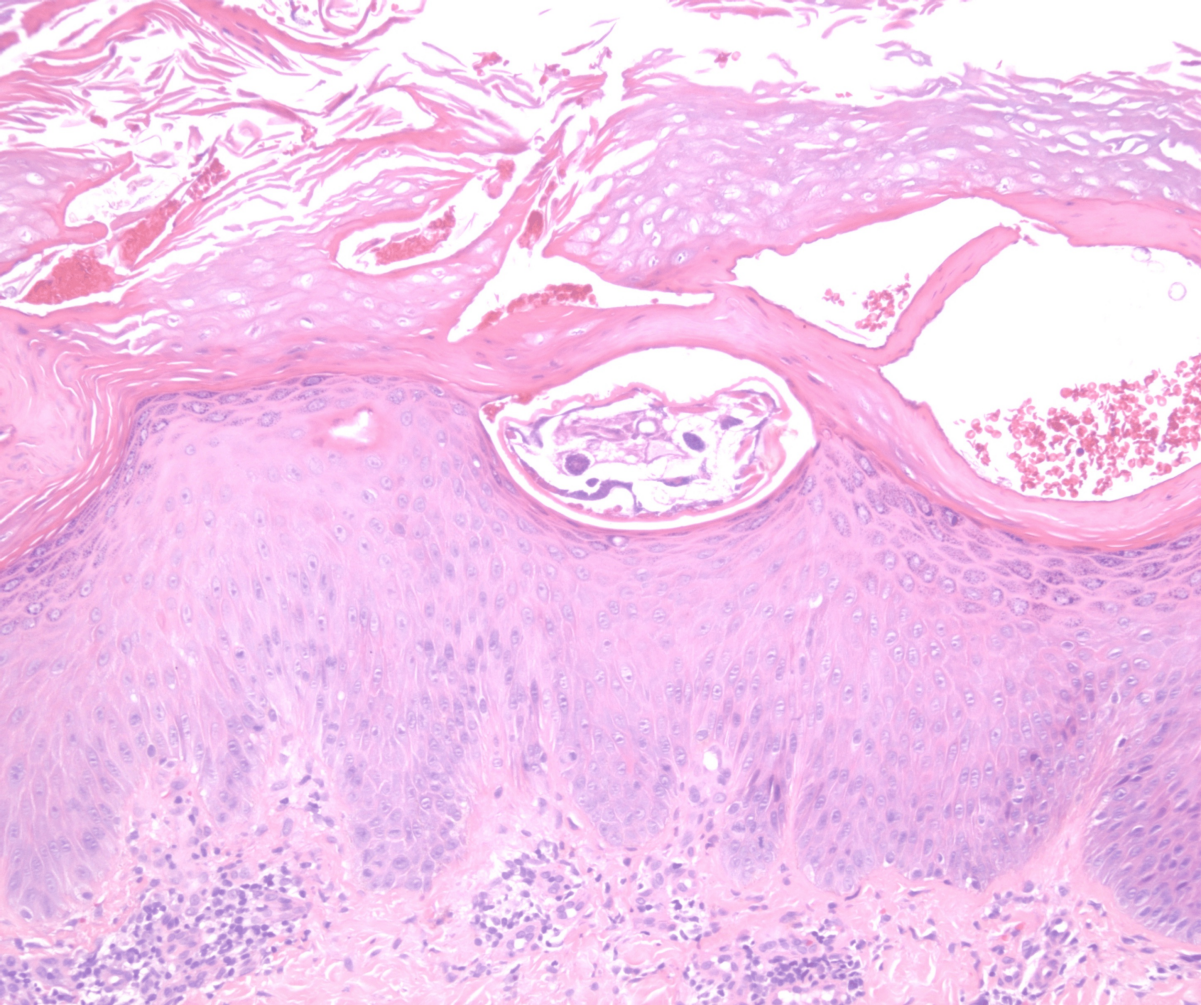
Histopathology demonstrated focal parakeratosis and a sarcoptic mite underlying the stratum corneum in the upper epidermis with superficial lymphocytic infiltrate and scattered eosinophils.

A 22-year-old immunocompetent male with a past medical history of dyshidrotic hand eczema previously treated with dupilumab presented to our dermatology clinic in July 2024 with new areas of skin involvement and a morphology distinct from his prior eczema. He had first started dupilumab in August 2023 but developed a flare on the hands and feet within the first month, initially thought to represent psoriasis, leading to discontinuation of the medication in September 2023. In November 2023, several of his roommates were diagnosed with scabies, and he was empirically treated with ivermectin 15 mg once, followed by a second 15 mg dose one week later. His eruption resolved, and dupilumab 300 mg every two weeks was restarted in May 2024 for recurrent dermatitis. Approximately five weeks into this second course of dupilumab, he returned with a new non-pruritic rash. Physical examination was significant for multiple red-orange papules involving the buttocks, arms, and flanks and a new hyperkeratotic, yellow-orange plaque on the right elbow (Figures 1A-C). HIV, hepatitis, and HTLV-I/II serologies were negative. A biopsy of the right elbow (Figure 2) showed focal parakeratosis and a sarcoptic mite underlying the stratum corneum in the upper epidermis with superficial lymphocytic infiltrate and scattered eosinophils. He was treated with a seven-day dosing regimen of ivermectin, resulting in complete resolution (Figure 1D) that remained clear at one-year follow-up.

## Discussion

CS is Th2-mediated, characterized by increased Th2 cytokines (IL-4, IL-5 and IL-13), driving the clinical skin findings [2,17]. Dupilumab specifically binds to the IL-4Rα subunit, which is shared by the IL-4 and IL-13 receptor complexes, thus reducing the Th2 response [18]. In a patient on dupilumab, this Th2 suppression could lead to a shift toward a relative Th1 dominance. While this shift might reduce itching by suppressing the inflammatory pathways typically involved in scabies, it can impair the immune system’s ability to effectively control the scabies mites, as Th1 responses are less effective against extracellular parasites like the scabies mite [2,19]. Helminthic infestations have also been reported in patients receiving dupilumab, further supporting the link between Th2 blockade and increased susceptibility to parasitic infections [20]. Additionally, this suppression of Th2 pathways can mask the hallmark itching of scabies as seen in our case, potentially giving the false impression of disease resolution. As a result, an untreated infestation may persist, eventually allowing unchecked mite proliferation and the progression from OS to CS. Research involving the genotyping of individual mites from previously treated patients over a four-year period showed that sequential episodes of infestations were genetically closer to each other than to mites from other patients, suggesting incomplete clearance of the mites rather than environmental re-infection [21]. Therefore, patients taking dupilumab may required a seven-day extended regimen of anti-scabietic drugs to truly eradicate the infestation. Further research is needed to investigate the transition of the host immune response from a Th1 to a Th2 profile in scabietic infections. However, this case should raise awareness regarding the potential for recalcitrant scabietic infection in patients receiving dupilumab therapy.

## Conclusions

CS remains a rare but serious dermatologic condition, particularly in the context of immunomodulatory therapy. This case report is the first to document CS in patients on dupilumab requiring an extended seven-day regimen of ivermectin for complete resolution. These findings expand on prior reports by highlighting the potential for dupilumab-induced Th2 suppression to not only permit mite proliferation but also obscure classic scabies symptoms such as pruritus. Clinicians should maintain a high index of suspicion for CS in patients presenting with atypical hyperkeratotic eruptions during or following dupilumab therapy. Our case underscores the need for more aggressive and prolonged anti-scabietic treatment in this population and suggest that current treatment protocols may require adjustment in the setting of biologic therapy.

## References

[1] Micali G, Lacarrubba F, Verzì AE, Chosidow O, Schwartz RA (2016). Scabies: advances in noninvasive diagnosis. PLoS Negl Trop Dis.

[2] Bhat SA, Mounsey KE, Liu X, Walton SF (2017). Host immune responses to the itch mite, Sarcoptes scabiei, in humans. Parasit Vectors.

[3] Niode NJ, Adji A, Gazpers S (2022). Crusted scabies, a neglected tropical disease: case series and literature review. Infect Dis Rep.

[4] Goyal NN, Wong GA (2008). Psoriasis or crusted scabies. Clin Exp Dermatol.

[5] Chandler DJ, Fuller LC (2019). A review of scabies: an infestation more than skin deep. Dermatology.

[6] Chosidow O (2006). Clinical practices. Scabies. N Engl J Med.

[7] Ortega-Loayza AG, McCall CO, Nunley JR (2013). Crusted scabies and multiple dosages of ivermectin. J Drugs Dermatol.

[8] Workowski KA, Bachmann LH, Chan PA (2021). Sexually transmitted infections treatment guidelines, 2021. MMWR Recomm Rep.

[9] (2025). Centers for Disease Control and Prevention. Post-travel dermatologic conditions. CDC Yellow Book 2024: Health Information for International Travel. CDC Yellow Book.

[10] Salavastru CM, Chosidow O, Boffa MJ, Janier M, Tiplica GS (2017). European guideline for the management of scabies. J Eur Acad Dermatol Venereol.

[11] Wang MK, Chin-Yee B, Lo CK (2019). Crusted scabies in a renal transplant recipient treated with daily ivermectin: A case report and literature review. Transpl Infect Dis.

[12] Roberts LJ, Huffam SE, Walton SF, Currie BJ (2005). Crusted scabies: clinical and immunological findings in seventy-eight patients and a review of the literature. J Infect.

[13] Davis JS, McGloughlin S, Tong SY, Walton SF, Currie BJ (2013). A novel clinical grading scale to guide the management of crusted scabies. PLoS Negl Trop Dis.

[14] Hasan T, Krause VL, James C, Currie BJ (2020). Crusted scabies; a 2-year prospective study from the Northern Territory of Australia. PLoS Negl Trop Dis.

[15] Bergamin G, Hudson J, Currie BJ, Mounsey KE (2024). A systematic review of immunosuppressive risk factors and comorbidities associated with the development of crusted scabies. Int J Infect Dis.

[16] Chong D, Sheka D, Mukovozov I (2024). Development of crusted scabies during biologic therapy: a systematic review. Mendeley Data.

[17] Walton SF, Pizzutto S, Slender A (2010). Increased allergic immune response to Sarcoptes scabiei antigens in crusted versus ordinary scabies. Clin Vaccine Immunol.

[18] Walton SF (2010). immunology of susceptibility and resistance to scabies. Parasite Immunol.

[19] Jankovic D, Sher A, Yap G (2001). Th1/Th2 effector choice in parasitic infection: decision making by committee. Curr Opin Immunol.

[20] Tan LD, Schaeffer B, Alismail A (2019). Parasitic (helminthic) infection while on asthma biologic treatment: not everything is what it seems. J Asthma Allergy.

[21] Walton SF, McBroom J, Mathews JD, Kemp DJ, Currie BJ (1999). Crusted scabies: a molecular analysis of Sarcoptes scabiei variety hominis populations from patients with repeated infestations. Clin Infect Dis.

